# Clear cell meningiomas—case presentation, review of radiographic identifiers, and treatment approaches

**DOI:** 10.1007/s00381-024-06390-z

**Published:** 2024-04-18

**Authors:** Margaret Keymakh, Joshua A. Benton, Rose Fluss, Seyed Ahmad Naseri Alavi, Allison M. Martin, Steven Chin, Andrew J. Kobets

**Affiliations:** 1 The Leo M. Davidoff Department of Neurological Surgery, Montefiore Medical Center, Albert Einstein College of Medicine, 3316 Rochambeau Avenue, 2nd Floor, Bronx, NY 10467 USA; 2 Department of Pediatrics, Albert Einstein College of Medicine and Division of Pediatric Hematology, Oncology and Cellular Therapy, Children’s Hospital at Montefiore, Bronx, NY USA; 3 Department of Pathology, Montefiore Medical Center, Albert Einstein College of Medicine, Bronx, NY USA

**Keywords:** Meningioma, Adjuvant therapy, Pediatric, Spine, Tumor

## Abstract

Spinal clear cell meningiomas (CCMs) are a rare histological subtype of meningiomas that pose preoperative diagnostic challenges due to their radiographic similarities with other lesions. They are also more aggressive, exhibiting higher rates of recurrence, particularly in pediatric patients. Overcoming diagnostic challenges of these tumors can improve patient outcomes. In this report, we describe a case of a pediatric patient presenting with a lumbar CCM in whom we were able to obtain gross total resection. Our report reviews previously identified predictors of CCM recurrence, including the Ki-67 proliferation index, number of spinal segments involved, and hormonal influences related to age and sex. We describe the characteristic radiographic features that differentiate spinal CCMs from other tumors to improve pre-operative diagnosis. Furthermore, we provide our rationale for adjuvant therapy for pediatric patients to refine treatment protocols for these rare tumors.

## Introduction

Meningiomas are primary central nervous system tumors, typically arising from the arachnoid cap cells of the meninges. Spinal meningiomas account for 1.2–12.7% of primary meningiomas and 4.3% of all intraspinal tumors [1, 2]. They are relatively rare in pediatric patients, representing 10% of pediatric meningiomas [3]. Among various meningioma subtypes, clear cell meningiomas (CCMs) are a World Health Organization (WHO) grade II meningioma. They exhibit unique histopathological features, clinical behavior, and diagnostic challenges that distinguish them from more common meningioma variants [4]. 

Composing only 0.2% of spinal meningiomas in the pediatric population [5], spinal CCMs are exceedingly rare. Their presentation depends on the location of the tumor, but patients often present with worsening bilateral lower extremity pain, weakness, and lower back pain. While these symptoms vary in severity, they can progress rapidly if the tumor is left untreated, leading to neurological deficits. The surgical management and long-term care of pediatric patients with spinal clear cell meningioma requires a specialized approach given the risks of adjuvant therapies, such as radiation for younger patient populations. Complications in diagnosing CCMs radiographically, as they have the appearance of more benign lesions without dural tails, make management even more complicated, and lead to misdiagnosis. Therefore, a thorough understanding of radiographic findings, as well as the natural history of these tumors, will allow for better care of these patients in the future.

Here, we present a case of a 17-year-old male with clear cell spinal meningioma that underwent gross total resection. By examining this case and supporting literature, we aim to shed light on the distinctive clinical considerations of this uncommon subtype, with a focus on patient-specific factors that are predictive for recurrence and development of an advantageous postoperative course. We also focus on the radiographic features which delineate these lesions from less aggressive intraspinal masses which may require different management strategies.

## Exemplary case description

**Fig. 1:**
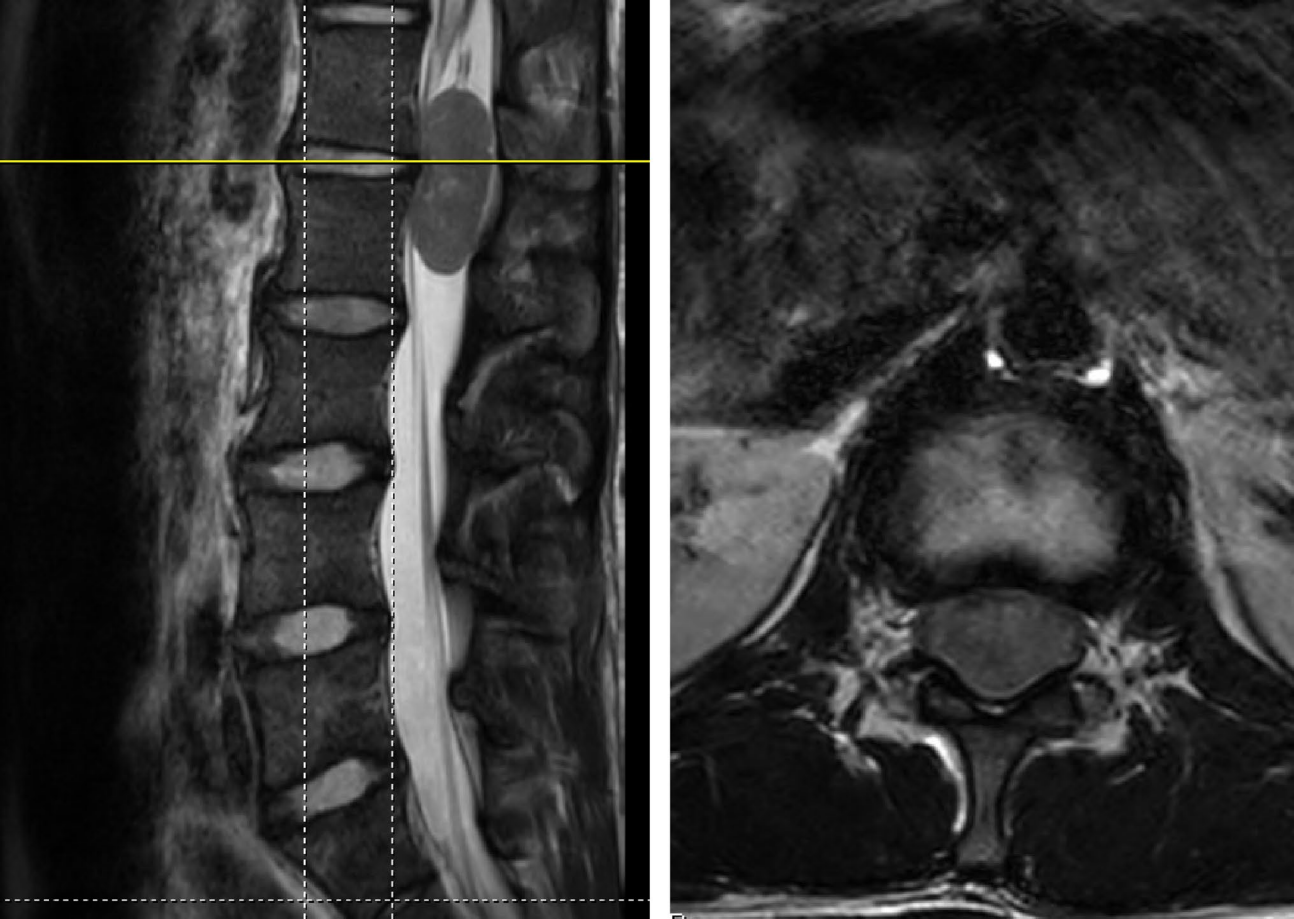
Preoperative MRI lumbar spine without contrast—T2 sagittal and axial sequences (left and right, respectively)—demonstrating an intradural extramedullary lesion centered between the L1-L2 vertebral bodies

A 17-year-old male with a history of mild scoliosis, presented with pain in the right buttock and thigh for one year. He had no specific preceding injury or trauma, noting pain in the morning and evening unrelated to activity. The pain was only in the buttock and posterior thigh but progressed to involve the anterior thigh; he had no other symptoms. An MRI of the lumbar spine was obtained for further evaluation, demonstrating an intradural, extramedullary lesion centered at L1-2 for which he was referred to pediatric neurosurgery (Fig. 1). The lesion was suspicious for a nerve sheath tumor given its appearance and lack of dural attachment. After discussion with the patient and family, the decision was made to proceed with surgery for resection of the lesion.

**Fig. 2:**
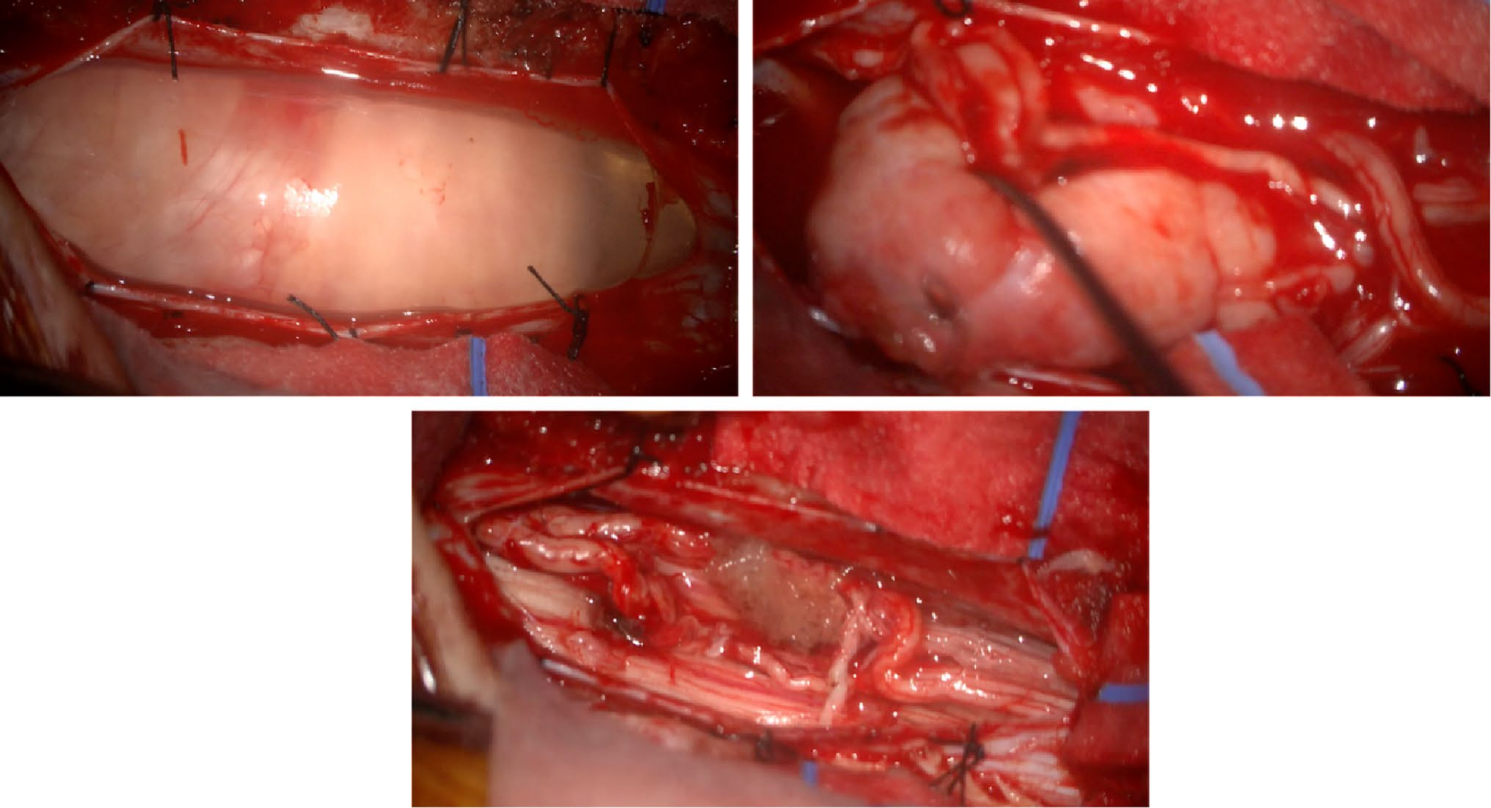
Intraoperative views. The top left and right images demonstrate the intradural lesion. The bottom image provides an intradural view after gross total resection of the mass

The patient underwent an L1 laminectomy with partial laminectomy of the caudal portion of T12 and cranial portion of L2 to adequately expose the cranial and caudal components of the lesion. The tumor was then identified on ultrasound to confirm adequate exposure. The dura was then opened, and the lesion was identified with several nerve roots splayed over a firm lesion with a thick capsule. The lesion was bound to a cord cranially and caudally which did not stimulate with 5 mAs of stimulation via a monopolar neuromonitoring electrode. Neuromonitoring was regularly checked given the intimate relationship between the tumor and adjacent nerve roots, thus minimizing the risk of nerve injury. After the frozen section was sent and the diagnosis confirmed, the capsule was opened, and the lesion was internally debulked to facilitate rotation and meticulous separation of adherent roots off the capsule to permit gross total resection (Fig. 2). Neuromonitoring remained stable throughout the case. The dura was then subsequently closed in watertight fashion, and the remainder of the tissue was closed in standard fashion without issue.

**Fig. 3:**
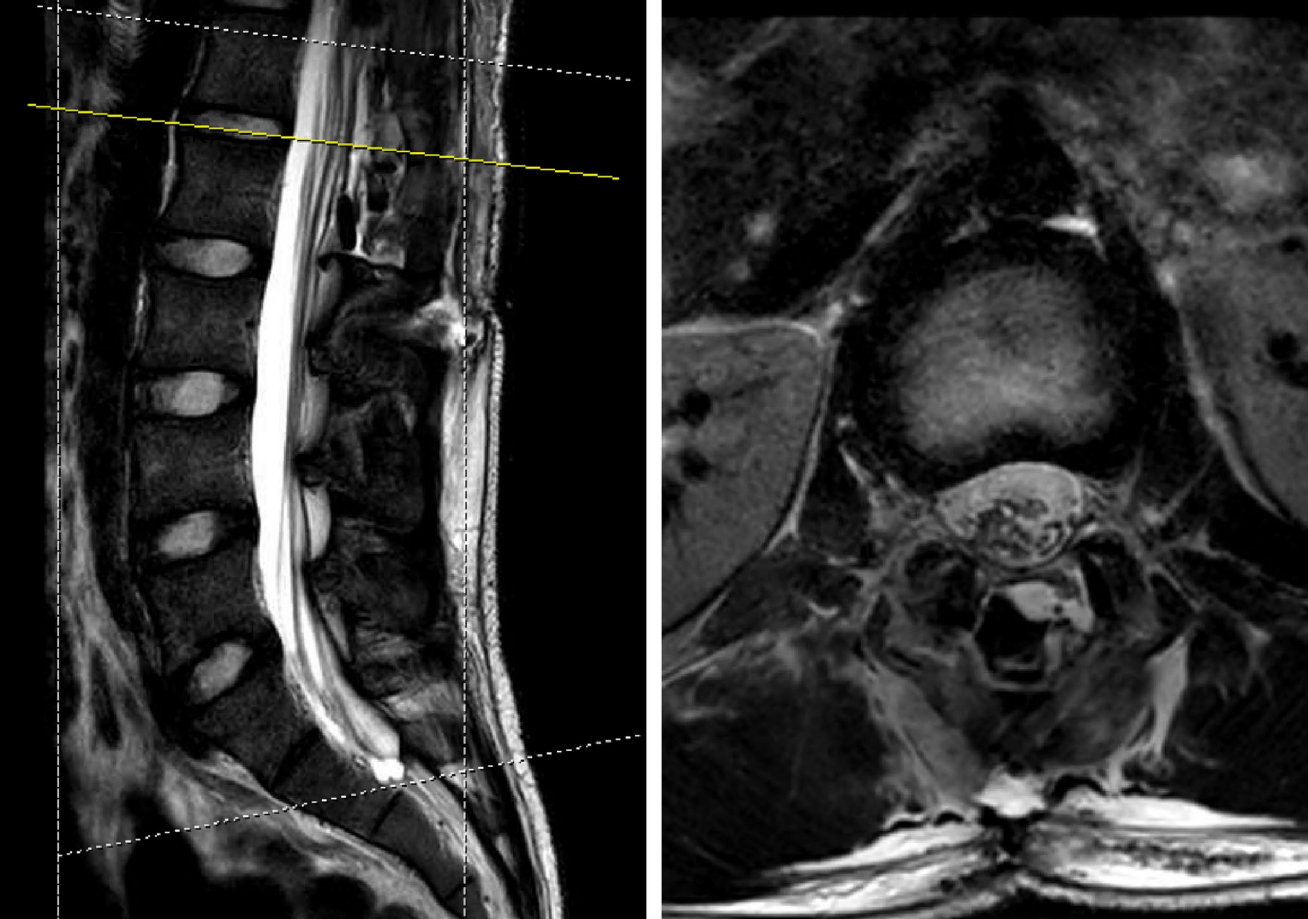
MRI lumbar spine without contrast obtained postoperative day 1. T2-weighted sagittal and axial images (left and right, respectively) demonstrating resection of lesion. The axial image is centered at the L1-L2 level

**Fig. 4:**
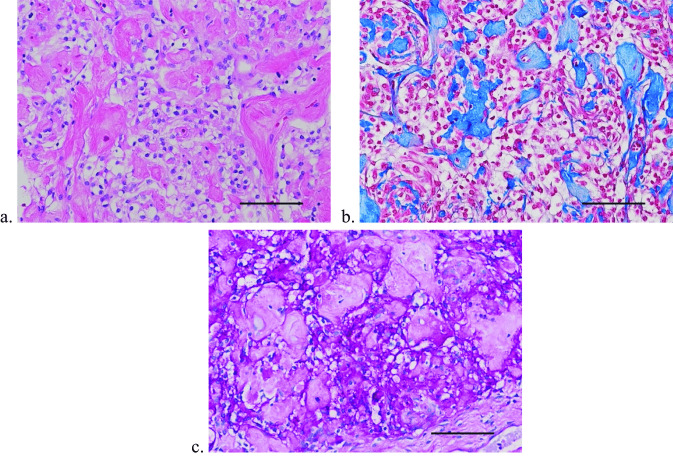
**a** The tumor cells exhibit characteristic optically clear cell cytoplasm and bland nuclei and are associated with aggregates of perivascular and interstitial collagen. Hematoxylin–eosin stain. Scale bar = 100 microns. **b** The aggregates of collagen are highlighted with Aniline Blue component of the Masson trichrome stain. Scale bar = 100 microns. **c** A periodic acid-Schiff histochemical stain shows the presence of glycogen granules within the cell cytoplasm of the tumor cells. Pretreatment with diastase confirmed the granular material as glycogen (not shown). Periodic acid-Schiff stain. Scale bar = 100 microns

Postoperatively, the patient was extubated and brought to the pediatric intensive care unit in stable condition. An MRI lumbar spine was obtained on postoperative day one, demonstrating gross total resection (Fig. 3); the rest of the spine was imaged as well, and no other lesions were identified. Postoperatively, the patient remained neurologically intact with resolution of his pain and paresthesias. He was discharged home postoperative day three. The final pathology returned as a clear cell meningioma (Fig. 4).

All data presented in this report was approved by the Albert Einstein College of Medicine/Montefiore Medical Center institutional review board (*Investigating outcomes in pediatric neurosurgical diseases*).

## Historical background

Clear cell meningioma, also known as glycogen-rich meningioma, was first described by Manivel and Sung in 1990 [6]. The most prominent histological feature of CCMs is the predominance of polygonal clear cells, which appear large and vacuolated due to the accumulation of glycogen granules [7]. Clear cells have a benign appearance, with round to oval nuclei, small nucleoli, and finely dispersed chromatin; however, they exhibit a more aggressive nature and have a higher rate of recurrence than other subtypes with reported recurrence rates of 23.8% [8].

## Diagnosis

Tissue specimens remain the gold-standard for diagnosing these lesions; however, preoperative imaging provides the initial diagnostic guidance. Surgeons must attempt to differentiate them from other similar lesions because they may masquerade as more benign pathologies. CCMs must be delineated from schwannomas and neurofibromas, as each is bonded to a single cord of fascicle and does not have the appearance of a dural tail. Both schwannomas and clear cell meningiomas may show contrast enhancement on imaging; CCMs often exhibit homogenous enhancement and about 25% of meningioma lesions contain calcifications [9]. Schwannomas may enhance, but their appearance is more variable. They might demonstrate a characteristic target sign—a central area with decreased signal intensity surrounded by rings of stronger enhancement [10].

Additionally, the margins and location of the lesions within the spinal canal can also help to elucidate diagnosis. Both CCMs and schwannomas typically present with clear, well-defined borders, although schwannomas are more likely to exhibit cystic areas or areas of degeneration [11]. On the other hand, neurofibromas tend to have less well-defined margins, due to their tendency to infiltrate surrounding tissues. This makes their margins less distinct on imaging [12]. Furthermore, CCMs and schwannomas favor the lumbar region of the spine, while neurofibromas are most typically found in the cervical spine [13]. Patient medical history is also relevant to diagnosis. Patients with neurofibromatosis are more likely to present with nerve sheath tumors rather than CCMs. Cutaneous stigmata must be evaluated in these suspected patients to frame the differential diagnosis of the spinal mass.

Utilizing a more refined approach to diagnosis utilizing subtle radiographic findings will aid the surgeon and the neuro-oncology team in their preoperative preparations and discussions with the family. Surgical resection will require a more aggressive approach to provide the patient with the best long-term prognosis. This approach will also help guide intraoperative disconnection of the lesion and general anticipation for separation from adjacent anatomical structures. Therefore, a thorough understanding of the radiographic and clinical findings of preoperative CCMs will facilitate safer and more comprehensive care for our patients.

## Management

The defined standard for treatment of clear cell meningiomas is gross total resection (GTR). Zhang et al. achieved GTR in 91.7% of patients with spinal CCMs, which exhibited a 20.8% rate of recurrence in comparison to 57.1% for subtotal resection [14]. Several other studies have come to similar conclusions, establishing the significance of extent of resection to postoperative outcomes [15–17].

No consensus exists on the necessity and effectiveness of adjuvant therapies for reducing post-operative recurrence of clear cell meningiomas. For intracranial CCMs, prior studies have affirmed the importance of postoperative radiotherapy after subtotal resection (STR), but it is not always suggested after a GTR, with largely inconclusive evidence for its efficacy [8]. Tao et al. recommend postoperative radiotherapy regardless of the extent of resection, since 9 patients received postoperative radiotherapy after GTR with no subsequent recurrence [18]. Another study assessed 99 patients that underwent GTR for intracranial CCMs and found no significant difference in recurrence-free survival with and without postoperative radiotherapy [14]. Furthermore, for patients receiving adjuvant radiotherapy after STR, radiation dosage did not show an association with overall survival [19].

Spinal CCMs show a reduced relapse rate after the first resection relative to intracranial tumors [20]; thus, adjuvant therapy for spinal CCMs is not usually advised with GTR. Overall, the rate of recurrence for intracranial CCMs is 63.3% and 40% for intraspinal CCMs [21]. Tao et al. concluded that radiotherapy should not be performed immediately following the first operation for spinal CCMs due to this significantly lower rate of recurrence in comparison to intracranial subtypes [18]. This difference in recurrence rate can also be explained by higher rates of GTR for spinal CCMs; given their rarity, the data available about recurrence may not reflect the true association between radiotherapy and post-operative outcomes. No significant difference in survival with adjuvant radiotherapy has been reported for spinal CCMs in comparison to GTR alone; however, post-operative radiotherapy after GTR is suggested by some authors due to potential reduction of recurrence for high-risk cases [22]. Therefore, we advocate for a watch-and-wait conservative approach after gross total resection. After surgery, 48-h and 3-month postoperative imaging should be obtained. If there is no evidence for recurrence on the immediate postoperative scan and the delayed 3-month study, we advocate for continued conservative therapy with serial imaging. Adjuvant radiation therapy should only be started with concern for nodular recurrence or the appearance of residual tumor.

### Prognosis and outcomes

Prior research has identified several predictors for outcomes with spinal CCMs. The Ki-67 proliferation index of the tumor has been suggested for use as a prognostic index for CCM recurrence. In a study with 13 patients, Zorludemir et al. found that the mean Ki-67 index for those that experienced recurrent tumors was significantly higher than those who did not [23]. The average Ki-67 index for recurrent patients was 13.3%, as compared to 7.4% for the non-recurrent group. Our patient’s tumor had a Ki-67 proliferation index of 4%. However, there have been instances of recurrence with a low Ki-67 index, as well as high Ki-67 cases without recurrence [21]. This shows that Ki-67 could have a predictive effect on prognosis, but it should not be considered as a clear signifier of future tumor development.

The number of spinal segments involved in spinal CCMs may predict recurrence. Spinal CCMs most often occur in the lumbar region, with about one-third of patients having two or more segments involved [20]. The proportion of patients with involvement of long segments spanning three or more levels has been reported up to 36.9%, in comparison to 2–11% for conventional spinal meningiomas [16]. Patients with these extended segmental involvements demonstrate a notably elevated recurrence rate [22]. Multi-segment involvement can have more complex anatomical features, thereby complicating surgical resection: the increased risk of residual tumor cells makes GTR harder to achieve.

The relationship between age, sex, and the recurrence of spinal clear cell meningiomas is likely mediated by the impact of hormonal influences on this histological type. While older adults have a higher risk of morbidity, age of ≤ 18 years old is a positive predictor of recurrence of spinal CCMs, with children exhibiting a threefold higher rate of recurrence [12]. There is less direct evidence of sex specificity, but it has been found that the female/male ratio of spinal CCMs is 1.7/1, indicating that there may be some female dominance [14, 24]. Given the previously established positive correlation between hormone replacement therapy and occurrence of meningiomas [25], it is hypothesized that some clear cell meningiomas may express growth or sex hormone receptors. Pediatric patients may be more susceptible to hormonal influences on tumor behavior because of the ongoing maturation of their endocrine systems. Likewise, higher levels of estrogen and progesterone in female patients may elicit tumor interactions that lead to recurrence [14]. These interactions are crucial for establishing sex and age-specific growth patterns.

The decision to offer pediatric patients with CCMs adjuvant radiotherapy requires balancing the increased risk of recurrence for younger patients with increased risk of side effects from radiotherapy treatments. In children, radiation has been shown to induce myelopathy, organ damage, and vertebral radiation necrosis with spinal cord dislocation [15, 17]. The choice to administer adjuvant therapies should be guided by the tumor’s specific characteristics including grade, histological features and location, and the patient’s overall health. Close postoperative monitoring with regular imaging is crucial for detecting early signs of recurrence. Therefore, we advocate for a watch-and-wait conservative approach after gross total resection, our operative goal. After surgery, 48-h and 3-month postoperative imaging should be obtained. If there is no evidence for recurrence on the immediate postoperative scan and the delayed 3-month study, we support continued conservative therapy with serial imaging. Imaging every 3–6 months is suggested for pediatric patients with spinal CCMs for the first several years after the operation, after which the interval can be increased to annually [17, 26]. Our patient underwent a follow-up MRI 3 months post-surgery and showed no sign of recurrent disease. Adjuvant radiation therapy should only be started with concern for nodular recurrence or the appearance of residual tumor. Ultimately, the decision should be made on a case-by-case basis to minimize the risk of recurrence while preserving overall well-being and quality of life for pediatric patients. Further studies on CCMs in pediatric patients should focus on long-term follow-up to provide insight on post-operative outcomes, recurrence rates, and late-onset complications. Their findings can help to guide refinement to treatment protocols and create clearer guidelines for adjuvant therapy.

## Conclusions

Clear cell meningiomas are a more aggressive meningioma subtype that tends to exhibit higher rates of recurrence in pediatric patients. While gross total resection remains at the gold standard of treatments, uncertainties persist regarding the necessity and effectiveness of adjuvant therapies. Factors including Ki-67 index, patient age, segmental involvement, extent of resection, and hormonal influences may help to establish recommendations for radiotherapy after surgery. Precise preoperative identification of clear cell meningiomas as distinct from other spinal lesions with similar radiographic findings will aid in tailored surgical approaches to optimize outcomes. We advise vigilant imaging for recurrence combined with a watch-and-wait approach in cases that achieve GTR. Further studies should prioritize long-term follow-up and analysis of more patient-specific factors to predict when recurrence is more likely and refine guidelines for adjuvant therapy. This will help to provide more comprehensive care for patients with these tumors and improve post-operative quality of life.

## Data Availability

No datasets were generated or analysed during the current study.
