# Correction: Clear cell meningiomas—case presentation, review of radiographic identifiers, and treatment approaches

**DOI:** 10.1007/s00381-024-06462-0

**Published:** 2024-05-29

**Authors:** Margaret Keymakh, Joshua A. Benton, Rose Fluss, Seyed Ahmad Naseri Alavi, Allison M. Martin, Steven Chin, Andrew J. Kobets

**Affiliations:** 1 The Leo M. Davidoff Department of Neurological Surgery, Montefiore Medical Center, Albert Einstein College of Medicine, 3316 Rochambeau Avenue, 2nd Floor, Bronx, NY 10467 USA; 2 Department of Pediatrics, Albert Einstein College of Medicine and Division of Pediatric Hematology, Oncology and Cellular Therapy, Children’s Hospital at Montefiore, Bronx, NY USA; 3 Department of Pathology, Montefiore Medical Center, Albert Einstein College of Medicine, Bronx, NY USA

**Correction to: Child's Nervous System** 10.1007/s00381-024-06390-z

In the Abstract sentence beginning 'Spinal clear cell meningiomas (CCMs) are a rare histological subtype of meningiomas that post preoperative diagnostic challenges due to their radiographic similarities with other lesions.' in this article, the text 'post' should have read 'pose'.

The original article has been corrected.

